# Utility of uneven double-lumen catheter for difficult guidewire manipulation in endoscopic ultrasonography-guided pancreaticogastrostomy

**DOI:** 10.1055/a-2318-2938

**Published:** 2024-07-04

**Authors:** Ayaka Machida, Yusuke Takasaki, Sho Takahashi, Akinori Suzuki, Shigeto Ishii, Toshio Fujisawa, Hiroyuki Isayama

**Affiliations:** 1 73362 Department of Gastroenterology, Juntendo University School of Medicine Graduate School of Medicine, Tokyo, Japan

Endoscopic ultrasonography-guided pancreaticogastrostomy (EUS-PGS) is a challenging procedure, and the most difficult step is guidewire insertion to the appropriate portion. We report herein a case in which the guidewire was not advanced in the correct direction after puncture during EUS-PGS, and a double-lumen catheter was used to correct the direction of the guidewire.

**Fig. 1:**
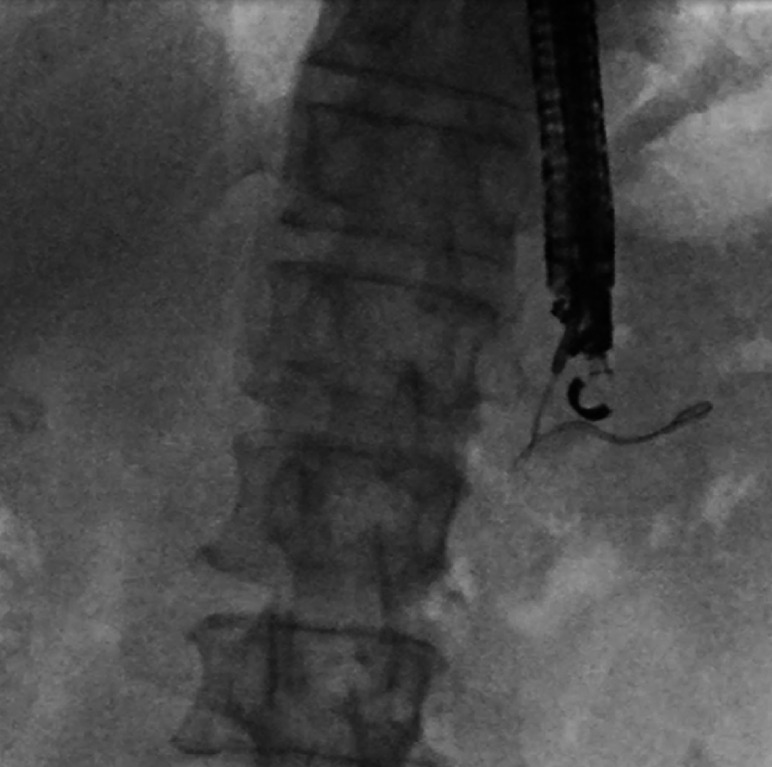
We were unable to insert the guidewire into the anastomotic site and, instead, could only place it on the opposite side (tail of the main pancreatic duct).

**Fig. 2:**
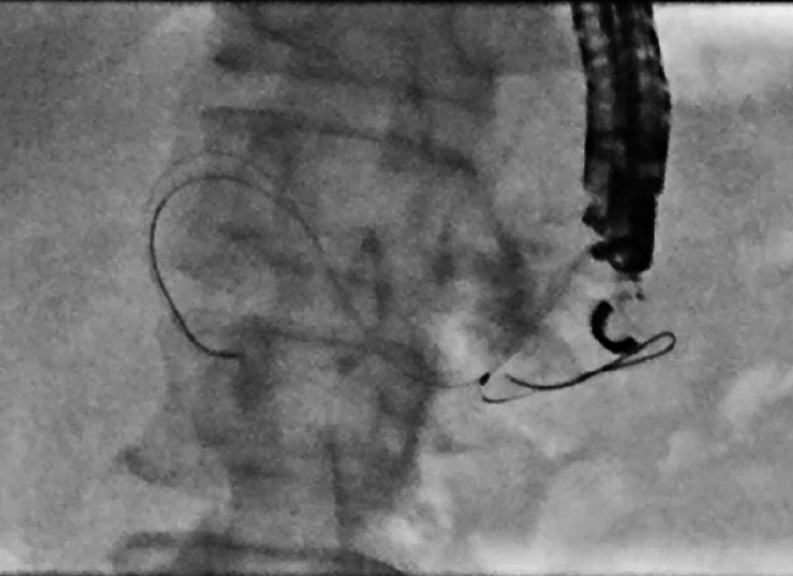
An uneven double-lumen cannula (Piolax Medical Devices, Yokohama, Japan) was inserted. A 0.025-inch guidewire (EndoSelector; Boston Scientific Japan, Tokyo, Japan) was then added from the other lumen and successfully inserted into the anastomotic site.

**Fig. 3:**
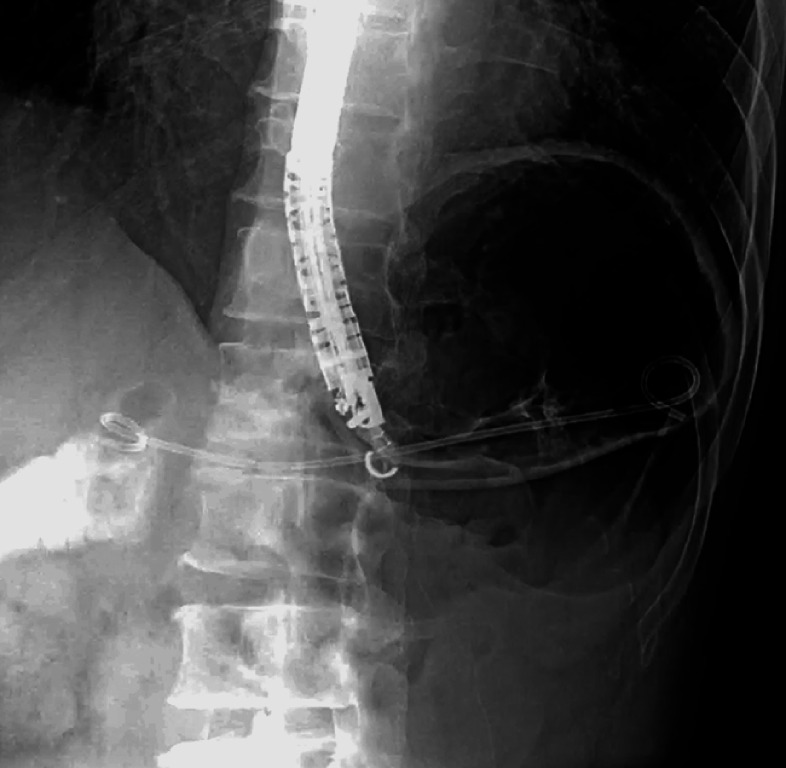
A 7-Fr, 15-cm, double-pigtail stent (Zimmon Biliary Stent; Cook Medical, Bloomington, Indiana, USA) was placed across the pancreaticojejunostomy stenosis.

A 57-year-old man who had undergone subtotal stomach-preserving pancreaticoduodenectomy 5 years previously was referred to our department for management of recurrent pancreatitis due to pancreaticojejunostomy stenosis (PJS). As pancreatic access with balloon-assisted endoscopy had failed at the previous institution, we performed EUS-PGS with antegrade stenting across the PJS. We successfully punctured the main pancreatic duct (MPD) from the stomach with a 19-gauge needle (EZ Shot 3 Plus; Olympus Medical Systems, Tokyo, Japan) and injected contrast medium. A 0.025-inch angled guidewire (VisiGlide 2; Olympus Medical Systems) was then placed in the tail of the MPD because insertion toward the anastomotic site had failed ( Fig. 1 ). We kept the guidewire in the MPD and dilated the puncture tract with a bougie dilator (ES Dilator; Zeon Medical, Tokyo, Japan). We inserted an uneven double-lumen cannula (Piolax Medical Devices, Yokohama, Japan) with an additional a 0.025-inch guidewire (EndoSelector; Boston Scientific Japan, Tokyo, Japan) in the other lumen. The additional guidewire was inserted into the opposite side and then passed across the PJS ( Fig. 2 ). The PJS and puncture tract were dilated with a balloon dilator (REN 4 mm; Kaneka, Tokyo, Japan). Finally, a 7-Fr, 15-cm, double-pigtail stent (Zimmon Biliary Stent; Cook Medical, Bloomington, Indiana, USA) was placed across the PJS ( Fig. 3 , Video 1 ).

When the guidewire was not advanced in the correct direction after puncture during endoscopic ultrasonography-guided pancreaticogastrostomy , a double-lumen catheter was useful for correcting the direction of the guidewire.Video 1

A double-lumen catheter was useful for changing the guidewire to the opposite side during EUS-PGS when the guidewire was not oriented in the correct direction.

Endoscopy_UCTN_Code_TTT_1AS_2AI

